# Title: Identifying Neuropsychiatric Symptoms in Lewy Body Dementia: A Revealing Case Report

**DOI:** 10.1192/j.eurpsy.2025.1677

**Published:** 2025-08-26

**Authors:** S. Aouadi, H. Ghabi, Y. Rebei, M. Karoui, H. Nefzi, R. Kammoun, F. Ellouze

**Affiliations:** 1 Psychiatry G , Razi Hospital; 2 Faculty of Medecine of Tunis, El Manar university, Tunis, Tunisia

## Abstract

**Introduction:**

Lewy body dementia (LBD) is characterized by a range of complex neuropsychiatric symptoms that can initially mimic other psychiatric disorders, particularly melancholic depression. Failure to recognize these symptoms can lead to delayed diagnosis and inappropriate management.

**Objectives:**

This case report highlights the importance of a thorough evaluation of neuropsychiatric symptoms in the diagnosis of Lewy body dementia (LBD).

**Methods:**

This case report was compiled through clinical observations, patient history from family members, and medical testing. Literature on LBD was reviewed to assess treatment strategies in light of this patient’s condition.

**Results:**

Mr. T.C., 75 years old, followed for hyperthyroidism and benign prostatic hyperplasia, was admitted for a severe depressive episode with melancholic features. Despite treatment with fluoxetine, mirtazapine, and olanzapine, his clinical condition did not improve. The patient exhibited food refusal, active suicidal ideation, and thoughts of incurability, along with a delusional syndrome. The Mini Mental State Examination (MMSE) revealed cognitive impairment with a score of 21/30. An initial brain MRI showed no abnormalities. The treatment was adjusted with the introduction of paroxetine alongside olanzapine and mirtazapine, but there was no significant improvement. A follow-up brain MRI revealed periventricular vascular leukoencephalopathy and moderate cortical atrophy, directing the diagnosis towards Lewy body dementia. The appearance of additional neuropsychiatric symptoms, including visual hallucinations, cognitive fluctuations, and mild parkinsonian signs, further supported the diagnosis of LBD. Treatment was then adjusted with the introduction of quetiapine, which is better tolerated in the context of LBD due to its favorable therapeutic profile.

**Conclusions:**

This case emphasizes the importance of accurately diagnosing neuropsychiatric disorders in Lewy body dementia. Appropriate management, based on a thorough clinical evaluation, can prevent treatment errors and improve the patient’s quality of life.

**Disclosure of Interest:**

None Declared

